# The Effect of Shift Work and Poor Sleep on Self-Reported Skin Conditions: A Survey of Call Center Agents in the Philippines

**DOI:** 10.3390/clockssleep1020023

**Published:** 2019-05-17

**Authors:** Francine Lu, Amanda Suggs, Harib H. Ezaldein, Jason Ya, Pingfu Fu, Jasmin Jamora, Vermen Verallo-Rowel, Elma D. Baron

**Affiliations:** 1 Skin and Cancer Foundation, Unit 1611, Medical Plaza Ortigas, Manila, Philippines; 2 Department of Dermatology, University Hospitals Cleveland Medical Center, Case Western Reserve University, Cleveland, OH 44106, USA; 3 Cleveland VA Medical Center, Cleveland, OH 44106, USA

**Keywords:** skin disease, sleep, circadian rhythm, acne, seborrheic dermatitis, atopic dermatitis

## Abstract

Night shift workers may have a disrupted circadian rhythm, which may contribute to the development of skin disease. The purpose of this study was to determine whether there is a significant difference in the prevalence and severity of self-reported skin disease between “regular” day shift workers compared to “graveyard” night shift workers. We conducted surveys from 630 call center agents in Manila, the Philippines, and they were analyzed regarding demographics, medical history, dermatologic history, lifestyle, and sleep. No difference was found in the prevalence of skin disease between shifts. However, night shift workers were worse sleepers. When compared to good sleepers, poor sleepers had a higher prevalence of skin disease with worse severity. Graveyard shift workers with poor sleep may have increased skin disease severity.

## 1. Introduction

The human body follows a circadian rhythm that is sensitive to external stimuli that, when disrupted, can affect the immune response and contribute to skin disorders, such as psoriasis, atopic dermatitis, and alopecia areata. It was observed that leukocyte counts peak during the night in humans, exhibiting diurnal variation in their ability to counter infections [1]. Li et al. suggested that night shift workers are at an increased risk of psoriasis, and that disruption of the circadian clock may contribute to its development [2].

Night shift workers, like call center agents, may have chronically disrupted circadian rhythms with chronic sleep deprivation and melatonin suppression by light exposure at night [3]. This career option remains very popular among Filipinos, with exceeding growth of the administrative and support service sector [4]. The primary aim of this study was to determine whether there is a significant difference in the prevalence and severity of self-reported skin disease between day shift workers compared to night shift workers. The secondary aim was to determine lifestyle habits and demographic factors that may predispose individuals to skin disease.

## 2. Results

### 2.1. Night versus Day Shift Workers

Seven hundred employees were surveyed. Of these, 70 were excluded: nine did not fulfill the criteria for age and 61 worked a given shift for less than one month. Of the 630 respondents analyzed, 41% (*n* = 259) worked day shift and 59% (*n* = 371) worked night shift. Night shift workers were older and had worked their shift for a longer duration. Though not significantly different, night shift workers had a higher BMI. Of the medical conditions surveyed, there was a significantly higher prevalence of hypertension among night shift workers compared to day shift workers (*p* = 0.043) (Table 1).

Among the dermatological conditions, there were no significant differences in the prevalence of self-reported skin (*p* = 0.716), hair (*p* = 0.557), or nail (*p* = 0.726) concerns between the two groups. The three most prevalent dermatological diseases among all respondents were acne vulgaris, dandruff/seborrheic dermatitis, and atopic dermatitis. There was no significant difference in prevalence of these dermatologic conditions between day and night shift workers. In terms of dermatological symptoms, there was no significant difference in itching (*p* = 0.494), dryness (*p* = 0.182), scaling (*p* = 0.073), or redness (*p* = 0.323) between the two groups.

Controlling the effects of sex, age, and BMI, both types of shifts were significant in predicting the overall SLIQ score. Compared to day shift employees, night shift workers had significantly lower overall SLIQ scores (*p* = 0.0001).

### 2.2. Good versus Poor Sleepers

The investigators also dichotomized the study population according to their Global PSQI scores, into either good sleepers or poor sleepers. More poor sleepers were overweight compared to good sleepers, though this was not significant (*p* = 0.056) (Table 2).

In terms of dermatological conditions, poor sleepers reported significantly more skin (*p* < 0.0001) concerns. There was no significant difference in hair (*p* = 0.55) and nail (*p* = 0.262) concerns. Acne (*p* = 0.0001), seborrheic dermatitis (*p* = 0.002), and atopic dermatitis (*p* = 0.027) were significantly more prevalent among poor sleepers. There was no significant difference in psoriasis incidence (*p* = 0.944). Skin redness (*p* = 0.136) and scaling (*p* = 0.433) were not significantly different. The incidence of itching (*p* = 0.002) and skin dryness (*p* = 0.0005) were significantly different between the two groups (Table 3).

The percentage of poor sleepers was higher in the night shift worker cohort (64.4%) compared to the day shift worker cohort (54.8%). There was no significant difference between the overall SLIQ scores between the regular or day shift (mean = 4.87, SD = 2.441) employees and graveyard or night shift (mean = 4.64, SD = 2.261) employees (*p* = 0.238). Both groups were reported to have a relatively unhealthy lifestyle (SLIQ = 0-4); however, when the SLIQ was broken down into its components, there was a significant difference between regular and graveyard shift employees in terms of smoking and stress, with more smokers and higher level of stress among the graveyard shift employees (Table 4). In terms of overall sleeping habits, both graveyard shift and regular shift scored poorly (global PSQI scores > 5), but the scores of the graveyard shift were significantly higher (mean = 6.81, SD = 4.212; *p* = 0.004) than the regular shift (mean = 5.83, SD = 3.612).

Controlling the effects of sex, age, and BMI, sleep was significant in predicting the overall SLIQ score. Good sleepers had lower SLIQ scores compared to poor sleepers (*p* < 0.001).

## 3. Discussion

A plausible explanation as to why employees choose to work at night may be sourced to Article 86 of the Philippine Labor Code, which entitles workers to at least 10% more per hour of work performed compared to morning or day shifts [5]. Overall, no significant difference was found between day and night shift workers in terms of skin, hair, or nail complaints. There was also no significant difference in dermatologic diseases. Interestingly, when the PSQI was broken down into its components, regular shift employees reported significantly better sleep quality, sleep latency, and sleep efficiency, but graveyard shift employees reported significantly longer sleep duration.

In this study, we chose to utilize patient self-reported outcomes as a measure of dermatological diseases and symptoms. Even outside of dermatology, recent initiatives have advocated for the use of patient-reported outcome measures, with the goals of both evaluating provider performance and improving patient outcomes [6]. Specifically, patient self-reporting has been validated in dermatology for identifying dermatological disease and identify symptoms for acne [7,8], psoriasis [9,10], and other skin conditions in a variety of study populations [11].

Hypertension was significantly more prevalent in graveyard shift workers. Hypertension is a risk factor for cardiovascular disease. As previously noted, there was a higher percentage of poor sleepers among graveyard shift workers. Other studies have also found a link between increased cardiovascular risk and poor sleep [12,13,14].

Regarding dermatologic disease, poor sleepers had significantly more skin concerns compared to good sleepers. There was no significant difference in hair and nail concerns between the two groups. Reports of acne vulgaris, seborrheic dermatitis and atopic dermatitis, skin dryness, and pruritic symptoms were more prevalent in poor sleepers. Poor sleep may potentiate the effects of stress on the skin. Sleep disorders, such as insomnia, have been found to be an important source of distress in patients with chronic skin disease [15]. Others studies have also shown a link between poor sleep and inflammatory dermatoses, such as acne vulgaris [16,17], atopic dermatitis [18,19], and psoriasis [15,20].

Limitations to our study include external validity of an analysis of call center agents based in Manila, Philippines, given differences in lifestyle and culture. The impact of graveyard shift work on health and skin disease may also differ by profession. The data was self-reported, possessing a recall bias that may under or overestimate the true severity of the disease process. Future studies are needed to evaluate the effect of circadian dysfunction on skin disease.

Overall, night shift workers with poor sleep may have more severe skin disease. Poor sleep may contribute to higher prevalence and more severe skin disease.

## 4. Materials and Methods

### Study Design

The study was approved by the Institutional Review Board (IRB) at Case Western Reserve University. The study complied with the ethical principles contained within the Declaration of Helsinki and the National Guidelines for Biomedical/Behavioral Research of the National Ethics Committee (NEC) of the Philippines.

The study was conducted at call center/business process outsourcing agents from multinational companies. Consent was obtained from the human resource department of each company involved.

Participants were surveyed regarding demographics (e.g., age, gender), height, weight, medical history, regular/graveyard shift work, and dermatologic history. The Simple Lifestyle Indicator Questionnaire (SLIQ) [21] and Pittsburgh Sleep Quality Index (PSQI) [22] were administered to each participant. The SLIQ assesses diet, physical activity, smoking, alcohol consumption, and stress.

The sample size was calculated using the OpenEpi calculator [23], based on the estimated population of call center agents in 2014 of 686,000 [24], with a confidence interval of 5, anticipated frequency of 50% and a 95% confidence level, leading to a sample size of 384.

Workers between the ages of 20–60 were included in the study. Per the literature [1], the diagnosis of shift work disorder can only be made if a patient has experienced symptoms for at least one month. Thus, participants who worked a certain shift for less than one month were excluded from the study. Day shift workers were considered those agents who started their shift between 6:00 a.m.–10:00 a.m. and ended their shift between 3:00 p.m.–7:00 p.m. Night shift workers started their shift anytime between 6:00 p.m.–10:00 p.m. and ended their shift between 3:00 a.m.–7:00 a.m. Patients with incomplete demographics (such as age) were also excluded. Using the results of the PSQI, patients were categorized as good sleepers (global PSQI = 5 or less) or poor sleepers (global PSQI > 5).

Descriptive statistics, mean and count (%), were used to present continuous and categorical variables. T-tests were used for comparisons involving continuous variables, such as age, weight, BMI, duration in a certain shift, and severity of skin condition. Chi-square analysis was done to compare categorical variables, such as gender, prevalence of medical and dermatological conditions, SLIQ scores, and PSQI scores. Data encoding and analysis were performed using Microsoft Excel and SPSS version 13. Data from participants who incompletely answered forms but still fulfilled the inclusion criteria were also included in the analysis, so as not to lose valuable data. *p* values less than 0.05 were considered statistically significant.

Study participants were given samples of skin products and discount coupons for their participation. Confidentiality of the subjects was maintained throughout the duration of the study. Only the investigators had access to the data contained within the survey forms.

## Figures and Tables

**Table 1:** Demographic profile of the respondents by shift.

	Regular/Day Shift (*n* = 259)		Graveyard/Night Shift (*n* = 371)		Significance
	Mean	SD ^†^	Mean	SD	
Age	27.14	6.037	29.11	5.582	<0.001
BMI	24.24	5.717	25.03	6.037	0.117
Hypertension prevalence	6.1		13.5		0.043
Duration of shift (months)	17.30	18.549	25.35	29.153	<0.001
	**%**		**%**		**Significance**
Male	50.6		46.1		0.152

^†^ SD = Standard deviation.

**Table 2:** Demographic profile of good vs. poor sleepers.

	Good Sleepers (*n* = 207)		Poor Sleepers (*n* = 423)		Significance
	Mean	SD	Mean	SD	
Age	28.42	7.062	28.23	5.163	0.736
BMI	24.07	6.055	25.02	6.264	0.056
Duration of shift (months)	20.91	23.642	22.60	26.569	0.420
Global PSQI score	2.28	2.115	8.93	2.379	<0.001
	**%**		**%**		**Significance**
Male	46.9		48.5		0.385

**Table 3:** Reported dermatologic conditions by demographic and shift features.

Dermatologic Condition		Shift (Graveyard vs. Regular)	Sleep (Good vs. Poor)	Sex (Female vs. Male)	Age (Per Year Increase)	BMI (Per Index Increase)
Psoriasis	**OR (95% CI)^£^**	0.83 (0.13, 5.13)	0.94 (0.15, 5.97)	0.62 (0.1, 3.94)	1.12 (1.01, 1.24)	0.84 (0.55, 1.27)
	***p*-value**	0.839	0.944	0.615	0.032	0.402
Dry skin	**OR (95% CI)^£^**	1.36 (0.87, 2.13)	0.4 (0.24, 0.67)	1.01 (0.66, 1.54)	1.03 (0.99, 1.07)	0.99 (0.91, 1.07)
	***p*-value**	0.182	0.0005	0.974	0.099	0.78
Itching	**OR (95% CI)^£^**	0.85 (0.54, 1.35)	0.41 (0.24, 0.71)	1.38 (0.87, 2.17)	1.02 (0.98, 1.06)	1.03 (0.95, 1.11)
	***p*-value**	0.494	0.002	0.172	0.308	0.499
Scaling	**OR (95% CI)^£^**	2.22 (0.93, 5.32)	0.71 (0.31, 1.66)	0.68 (0.32, 1.43)	1 (0.93, 1.07)	1.08 (0.96, 1.21)
	***p*-value**	0.073	0.433	0.306	0.95	0.226
Skin redness	**OR (95% CI)^£^**	1.44 (0.7, 2.96)	0.54 (0.24, 1.22)	0.9 (0.46, 1.75)	0.97 (0.9, 1.04)	1.1 (0.99, 1.22)
	***p*-value**	0.323	0.136	0.755	0.355	0.071
Skin asthma	**OR (95% CI)^£^**	1.32 (0.67, 2.6)	0.41 (0.18, 0.9)	1.34 (0.71, 2.55)	1 (0.94, 1.06)	0.93 (0.81, 1.06)
	***p*-value**	0.417	0.027	0.367	0.99	0.284
Dandruff/seborrheic dermatitis	**OR (95% CI)^£^**	0.8 (0.53, 1.2)	0.48 (0.3, 0.76)	1.51 (1.01, 2.26)	1.02 (0.99, 1.06)	1.03 (0.96, 1.11)
	***p*-value**	0.277	0.002	0.044	0.188	0.437
Acne	**OR (95% CI)^£^**	0.97 (0.67, 1.4)	0.47 (0.32, 0.69)	1.16 (0.81, 1.66)	0.96 (0.92, 0.99)	0.98 (0.92, 1.05)
	***p*-value**	0.866	0.0001	0.416	0.011	0.634
Unspecified nail concerns	**OR (95% CI)^£^**	0.76 (0.16, 3.61)	0.29 (0.03, 2.52)	4.96 (0.58, 42.74)	1 (0.85, 1.17)	0.7 (0.42, 1.16)
	***p*-value**	0.726	0.262	0.145	0.962	0.163
Unspecified hair concerns	**OR (95% CI)^£^**	1.35 (0.5, 3.69)	0.74 (0.28, 1.97)	2 (0.78, 5.15)	0.96 (0.87, 1.06)	1.04 (0.89, 1.21)
	***p*-value**	0.557	0.55	0.149	0.395	0.626
General skin concerns	**OR (95% CI)^£^**	0.93 (0.64, 1.35)	0.35 (0.24, 0.5)	1.04 (0.73, 1.49)	1 (0.96, 1.03)	1.03 (0.96, 1.1)
	***p*-value**	0.716	<0.0001	0.84	0.755	0.455

Highlighted cells for significant *p*-values.

**Table 4:** Simple Lifestyle Indicator Questionnaire (SLIQ) and Pittsburgh Sleep Quality Index (PSQI) component scores (% with score of 0 in category) by shift.

	Regular Shift	Graveyard Shift	Significance
**SLIQ Component scores by shift**			
Diet	59.1	64.2	0.396
Physical activity	19.3	27.0	0.63
Alcohol	0.4	2.7	0.072
Smoking	19.3	28.8	0.009
Stress	15.8	29.1	0.001
**PSQI Component Scores by Shift**			
Sleep quality	8.1	6.7	0.040
Sleep latency	12.4	8.9	0.001
Sleep duration	15.1	17.5	0.000
Sleep efficiency	60.2	37.2	0.000
Sleep disturbances	3.5	2.4	0.340
Use of sleep meds	78.0	76.5	0.126
Daytime dysfunction	21.6	17.8	0.450
